# Azithromycin therapy reduces cardiac inflammation and mitigates adverse cardiac remodeling after myocardial infarction: Potential therapeutic targets in ischemic heart disease

**DOI:** 10.1371/journal.pone.0200474

**Published:** 2018-07-12

**Authors:** Ahmed Al-Darraji, Dalia Haydar, Lakshman Chelvarajan, Himi Tripathi, Bryana Levitan, Erhe Gao, Vincent J. Venditto, John C. Gensel, David J. Feola, Ahmed Abdel-Latif

**Affiliations:** 1 Gill Heart Institute and Division of Cardiovascular Medicine, University of Kentucky, Lexington, KY, United States of America; 2 College of Pharmacy, University of Kentucky, Lexington, KY, United States of America; 3 The Center for Translational Medicine, Temple University School of Medicine, Philadelphia, PA, United States of America; 4 Spinal Cord and Brain Injury Research Center, Department of Physiology, College of Medicine University of Kentucky, Lexington, KY, United States of America; 5 The Lexington VA Medical Center, Lexington, KY, United States of America; University of Louisville, UNITED STATES

## Abstract

**Introduction:**

Acute myocardial infarction (MI) is a primary cause of worldwide morbidity and mortality. Macrophages are fundamental components of post-MI inflammation. Pro-inflammatory macrophages can lead to adverse cardiac remodeling and heart failure while anti-inflammatory/reparative macrophages enhance tissue healing. Shifting the balance between pro-inflammatory and reparative macrophages post-MI is a novel therapeutic strategy. Azithromycin (AZM), a commonly used macrolide antibiotic, polarizes macrophages towards the anti-inflammatory phenotype, as shown in animal and human studies. We hypothesized that AZM modulates post-MI inflammation and improves cardiac recovery.

**Methods and results:**

Male WT mice (C57BL/6, 6–8 weeks old) were treated with either oral AZM (160 mg/kg/day) or vehicle (control) starting 3 days prior to MI and continued to day 7 post-MI. We observed a significant reduction in mortality with AZM therapy. AZM-treated mice showed a significant decrease in pro-inflammatory (CD45^+^/Ly6G^-^/F4-80^+^/CD86^+^) and increase in anti-inflammatory (CD45^+^/Ly6G^-^/F4-80^+^/CD206^+^) macrophages, decreasing the pro-inflammatory/anti-inflammatory macrophage ratio in the heart and peripheral blood as assessed by flow cytometry and immunohistochemistry. Macrophage changes were associated with a significant decline in pro- and increase in anti-inflammatory cytokines. Mechanistic studies confirmed the ability of AZM to shift macrophage response towards an anti-inflammatory state under hypoxia/reperfusion stress. Additionally, AZM treatment was associated with a distinct decrease in neutrophil count due to apoptosis, a known signal for shifting macrophages towards the anti-inflammatory phenotype. Finally, AZM treatment improved cardiac recovery, scar size, and angiogenesis.

**Conclusion:**

Azithromycin plays a cardioprotective role in the early phase post-MI through attenuating inflammation and enhancing cardiac recovery. Post-MI treatment and human translational studies are warranted to examine the therapeutic applications of AZM.

## Introduction

Acute myocardial infarction (MI) is a leading cause of mortality and morbidity in the western world [1]. MI provokes a profound coordinated inflammatory response, a process mediated by inflammatory bone marrow (BM) and peripheral blood (PB) cells, which has been linked to the development of end stage heart failure (HF), a highly frequent complication post-MI [1]. The peri-infarct zone demonstrates dynamic cellular changes with the infiltration of various inflammatory cells including neutrophils, monocytes, and macrophages [2]. Monocytes infiltrate the peri-infarct zone and differentiate into macrophages, which play an important role in the initial inflammatory as well as the following reparatory phases [3]. Two dominant patterns of macrophage activation are found: pro-inflammatory/classically activated macrophages (M1-like) and anti-inflammatory/alternatively activated/reparative macrophages (M2-like), with different cell markers and gene expression profiles [2]. In mice, the initial exaggerated inflammatory response may actually confer long-term harm because reductions in the initial recruitment of inflammatory monocytes reduce infarct size and prevent adverse cardiac remodeling [4, 5]. Pro-inflammatory macrophages trigger inflammation, damage of extra cellular matrix (ECM) [6], production of reactive oxygen/ nitrogen species and pro-inflammatory cytokines (IL-6, TNF-α, and IL-1β) [7]. In contrast, anti-inflammatory macrophages promote ECM repair, angiogenesis, and production of anti-inflammatory cytokines (IL-4, IL-10, and IL-13) [8, 9].

Azithromycin (AZM), a clinically approved macrolide antimicrobial agent, has an excellent safety profile in humans [10]. AZM modulates the inflammatory response through macrophage polarization towards the reparative state [11, 12], as demonstrated in models of inflammation and tissue injury such as spinal cord injury [13], lung infection [14], and stroke [15]. In these clinically relevant scenarios, AZM reduces the production of pro-inflammatory cytokines (IL-6 and IL-12) and increases that of anti-inflammatory cytokines (IL-10) [12]. Additionally, AZM significantly decreased the expression of iNOS and pro-inflammatory macrophage receptor (CCR7) while increasing arginase activity and anti-inflammatory macrophage receptors (MR and CD23) [12]. In an ischemic stroke model, which induces a similar sterile inflammatory response to MI, AZM shifted macrophages from the pro-inflammatory to the reparative state leading to inhibition of blood brain barrier injury and improvement in neurological recovery [15]. Likewise, in a retinal ischemia/reperfusion experimental model, AZM was protective against neuronal injury. This protection was attributed to the anti-inflammatory properties of AZM, as evidenced by the reduction in MMP-9/2 expression and activity [16]. In addition to its immunomodulatory properties, AZM is well tolerated, achieves a wide therapeutic index, and has well characterized pharmacokinetic and pharmacodynamic properties [10].

Here, we provide the first evidence that AZM reduces the inflammatory response and mediates cardioprotection against adverse cardiac remodeling and HF post-MI. AZM produces its positive effects through increasing neutrophil apoptosis and switching macrophage activation towards an anti-inflammatory phenotype. Our findings present an important step towards designing clinically relevant strategies to reduce the risk of cardiac remodeling and HF after MI.

## Materials and methods

**Fig 1:**
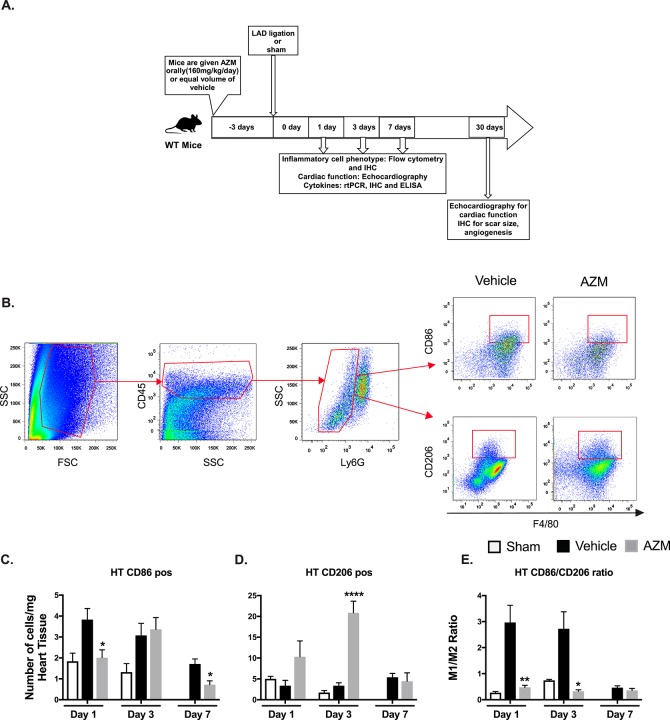
AZM therapy shifts macrophages towards the anti-inflammatory phenotype in the heart after myocardial infarction. Experimental design for the *in vivo* study (Panel A). Representative FACS plots demonstrating the gating strategy for pro-inflammatory (CD45^+^/Ly6G^+^/F4-80^+^/CD86^+^) and anti-inflammatory (CD45^+^/Ly6G^+^/F4-80^+^/CD206^+^) macrophages (Panel B). Quantitative analyses of pro-inflammatory subpopulations (CD86^+^) and anti-inflammatory subpopulations (CD206^+^) are presented in Panels C and D, respectively, at different time points following MI in AZM and vehicle treated groups. There is a significant reduction in the inflammatory macrophages in the first day after MI in AZM-treated mice, which is associated with a significant increase in anti-inflammatory ones. These changes translate into a significant reduction in the pro-/anti-inflammatory (CD86^+^/ CD206^+^) ratio at days 1 and 3 after MI (Panel E) (n = 4 MI and 3 sham mice/group/time point, *P<0.05, **P<0.01 and ****P<0.001 compared to vehicle controls). Data presented as mean ± SEM. AZM, azithromycin; IHC, immunohistochemistry; LAD, left anterior descending coronary artery; rtPCR, real-time Polymerase Chain Reaction.

Study Design. 8–10 weeks male C57BL/6 mice (Jackson Laboratory, BarHarbor, ME) were treated with either AZM (Azithromycin tablets, USP, Sandoze, NDC0781194133, Princeton, NJ), (crushed tablets suspended in 2% methylcellulose), orally using gastric gavage at 160 mg/kg/day or vehicle (2% methylcellulose), starting 3 days prior to MI or sham surgeries through day 7 post-MI (**Fig 1A**). Timing of AZM therapy was selected to ensure appropriate steady-state levels at the time of injury [14]. AZM administration was continued for 7 days after surgery to cover the entire duration of post-MI inflammatory response [17, 18]. AZM treatment was not associated with changes in liver or kidney function throughout the period of administration (**S1 Fig**). All procedures were conducted under the approval of the University of Kentucky IACUC in accordance with the NIH Guide for the Care and Use of Laboratory Animals (DHHS publication No. [NIH] 85–23, rev. 1996).

### Murine model of myocardial infarction

Mice were anesthetized with 1–3% isoflurane using a small animal vaporizer system. Pain reflexes were examined to make sure that the mouse is adequately anaesthetized prior to surgery. Following a left thoracotomy between the fourth and fifth ribs and removal of pericardial sac, the heart was exposed and pushed out of the thorax under direct visualization. The left anterior descending coronary artery (LAD) was identified under a surgical microscope and permanently ligated 3 mm below its origin using a 6–0 silk suture as previously described [19, 20]. The sham group underwent the same surgical procedure except the suture was passed under the LAD but not tied. Following LAD ligation, the heart was placed back into the intrathoracic space and the pneumothorax was manually evacuated. After muscle closure, the skin was sutured using 4–0 Prolene running sutures.

### Humane endpoints

Animals were treated with pain medications for 24–48 hours after surgery. This duration may be prolonged if animals showed signs of pain, discomfort or reduced food or water intake. Animals were followed every 8 hours for 24 hours followed by daily for 1 week, for any signs of clinical deterioration or overt heart failure. Their water and food consumption is monitored carefully. Any animals that show signs of decompensation were resuscitated and if this is unsuccessful, they were humanly euthanized. We also monitored mice for additional signs of distress or weight loss >15–20% from baseline weight; hunched posture; ruffled coat; etc. If the mice exhibited any of these signs, they were euthanized without any subsequent experimental procedures. Most of the mortality in our MI group occurred in the first week after surgery. Necropsy showed blood filled thoracic cavity suggesting myocardial rupture as the cause of death.

The appearance of the surgical site is checked for signs of infection, undue sutures, and seroma formation. When possible, infection, sutures, or seroma formation is treated medically (antibiotics, if indicated) or surgically (replacing sutures or percutaneous drainage, respectively). In rare cases in which infection appears to have occurred, we consulted with DLAR veterinary for advise, treatment and management. In cases where complications are felt to be causing the animal significant distress, the animal is euthanized.

### Method of euthanasia

Mice were euthanized using high dose isoflurane followed by cervical dislocation to confirm their euthanasia.

### Flow cytometry

Peripheral blood tubes containing 1:5 ratios of ethylene diaminetetraaceticacid (EDTA)/citrate-theophylline-adenosinedipyridamole (CTAD) were used to collect blood samples at 1, 3 and 7 days after MI (4 MI mice and 3 sham mice /treatment group). Whole blood was centrifuged at 700*g* for 5 minutes, and plasma layer was collected in separated tubes and kept at -80°C. The remaining cell pack was incubated with 0.5 ml of 1X of red blood lysing buffer (BD pharm lyse) for 10 minutes with gentle agitation for RBC lysis followed by dilution using 0.5 ml of staining buffer (5% goat serum, 0.05% sodium azide in phosphate-buffered saline) to stop the reaction. The suspension was centrifuged at 400*g* for 5 minutes, and supernatant was discarded. This step was repeated if we observed residual red blood cells. The white blood cell pellet was resuspended in staining buffer, washed to remove any remaining of lysis buffer, and centrifuged at 400*g* for 5 minutes. Cells from individual blood samples were split into two portions for flow cytometry and reverse transcription-polymerase chain reaction (RT-PCR) based on cell counts so that each sample contained ∼10^6^ live (trypan blue-negative) cells. Cells for rtPCR were lysed using cell lysis buffer (Life technologies) for 10 minutes with strong agitation and kept at -80°C for gene analyses. Cells for staining were immediately incubated on ice for 30 minutes with conjugated primary antibodies against FITC-conjugated Ly6G/C (BD Pharmingen), APC-CY7-conjugated CD45 (Biolegend), PE-conjugated CD115 (Biolegend) for the monocyte panel. After incubation, cells were washed twice using flow buffer and analyzed using an LSR II (Becton Dickinson) in the University of Kentucky Flow Cytometry Core. Monocytes were classified as pro-inflammatory (CD45^+^/CD115^hi^/Ly6-C^hi^) and anti-inflammaory (CD45^+^/CD115^hi^/Ly6-C^lo^). Neutrophils were identified as CD45^+^/CD115^lo^/Ly6G/C^lo^.

Heart tissue collected at the same time points were rapidly removed and placed in ice cold PBS (VWR International). The heart was manually minced using razor blades. Minced heart tissue was transferred into 15 ml tube with Collagenase B (Roche, Indianapolis, IN) and Dispase II (Roche, Indianapolis, IN) solution for 30 minutes in 37°C water bath, with manual agitation every 5–10 minutes. Then, the digestion reaction was terminated using cold staining buffer, and tubes were placed in ice. The digestion mixture was filtered through 70 μm cell strainers and centrifuged at 400*g* for 5 min at 4 ^0^C, then the supernatant was aspirated and cells were resuspended in 0.5 ml of staining buffer. Cells from individual hearts were split into approximately three portions based on cell counting so that each sample contained ∼10^6^ live (trypan blue-negative) cells for flow cytometry and RT-PCR as detailed above. Cells were incubated directly on ice for 30 minutes with conjugated primary antibodies against FITC-conjugated Ly6G (BD Pharmingen), PE-conjugated CD206 (Biolegend), PerCP-CY5.5-conjugated CD86 (Biolegend), PECY7-conjugated F4/80 (Biolegend), and APC-CY7-conjugated CD45 (Biolegend) for the macrophage panel, or the monocyte panel detailed above. After incubation, cells were washed twice using flow buffer and analyzed using an LSR II (Becton Dickinson). Macrophages were classified into pro-inflammatory (CD45^+^/Ly6G^-^/F4-80^+^/CD86^+^ cells) and ant-inflammatory (CD45^+^/Ly6G^-^/F4-80^+^/CD206^+^ cells). Laser calibration and compensation were carried out for every experiment utilizing unstained and single fluorescent controls.

Using the same protocols mentioned earlier, cells isolated from hearts and peripheral blood were incubated with APC-CY7-conjugated CD45 (Biolegend), FITC-conjugated Ly6G (BD Pharmingen), APC-conjugated Annexin 5 (Biolegend) and PE-conjugated propidium iodide (PI) (Biolegend) to identify apoptosis in neutrophils. Cells were considered apoptotic if they were PI^lo^/Annexin V^hi^. For flow analyses in all experiments we utilized FlowJo (version 7) software to generate dot plots and analyze the data.

### Histology

Mice (N = 12-15/treatment group) were sacrificed 30 days after MI and hearts were collected in diastole by using saturated KCl and CdCl_2_ (100 mM) given through the apex into the left ventricular (LV) cavity. Then we cannulated the ascending aorta and perfused the heart with PBS (VWR International) followed by 10% buffered formalin (VWR International) at 75 mmHg. Hearts were placed in 5 ml of 10% neutral buffered formalin (VWR) and fixed overnight at room temperature. Hearts were then sectioned into 2-mm cross-sectional slices followed by paraffin embedding. Tissue was transferred to 70% ethanol for overnight followed by sectioning into 4-μm sections starting at the level of LAD ligation. Sections were then stained with Masson’s trichrome to evaluate scar size. Digital images were taken, and areas were evaluated using NIH ImageJ (version 7). We measured the LV area, LV cavity area, and infarct area in stained sections, as previously described [19, 20]. The scar size was presented as a percentage of LV myocardial volume.

### Immunohistochemistry

Mice (N = 4/treatment group) were sacrificed 3 days after MI and hearts were collected. Immunohistochemical assessments were carried out on deparaffinized and rehydrated sections as previously described [20]. After deparaffinization and washing, slides were incubated with primary antibodies: rat anti-mouse CD86 (1:100, BD Biosciences), goat anti-mouse CD206 (1:1000, R and D Systems), rabbit anti-mouse YM1 (1:25, Stem Cell technologies), and goat anti-mouse IL-1β (1:200 R & D System) overnight at 4°C. After washing, sections were incubated with secondary antibodies conjugated to Alexa Fluor 594,647 (1:500, Invitrogen, Carlsbad, CA), then incubated with Sudan Black B (Sigma Aldrich, St. Louis, MO) for 30 minutes. and subsequently incubated with DAPI nuclear counterstain. 10–15 adjacent areas in the pre-infarct and remote zones per section were analyzed (1–2 sections/animal) at 40x magnification using Nikon Confocal Microscope A1 in the University of Kentucky Confocal Microscopy facility. Only nucleated antibody positive cells were counted. Calculations were performed using the Cell Counter plugin for ImageJ (version 1.51d). Data were presented as total positive cells per high power field in the region of interest.

A similar protocol was used to prepare heart sections from mice on day 30 for angiogenesis assessment using FITC- isolectin B4 (FL1201, Vector Labs, Burlingame, CA). 10–15 adjacent areas in the pre-infarct per section were examined (1–2 sections/animal) at 40x magnification using Nikon Confocal Microscope A1 in the University of Kentucky Confocal Microscopy facility. Data were presented as total capillary density per mm^2^ in the peri-infarct region. All measurements were analyzed by blinded observers.

To examine apoptosis in heart tissue, we used a TdT dUDP Nick-End Labeling (TUNEL) Assay and Caspase-3 staining. Caspase 3 staining was done in deparaffinized and rehydrated sections using antibody against cleaved caspase 3 as previously described [20]. TUNEL staining was performed in Biospecimen Procurement and Translational Pathology Shared Resource Facility (BPTP SRF) at the University of Kentucky. Only nucleated positive cells were counted in the peri-infarcted and infarcted areas. Calculations were done using the Cell Counter plugin for ImageJ (version 1.51d). Data were presented as total positive cells per high power field in the region of interest. All measurements were analyzed by blinded observers.

### Real-time polymerase chain reaction

We utilized PureLink RNA Mini Kit (ThermoFisher Scientific) to collect total RNA from heart and blood cells according to the manufacturer’s protocol. The extracted RNA was quantified using NanoDrop 8000 spectrophotometer (Thermofisher). Then, cDNA was synthesized using SuperScript VILO cDNA synthesis kit (Invitrogen). Quantitative RT-PCR was performed using a QuantaStudio 7 Flex real-time thermocycler (Applied Biosystems by life technology) to quantify the mRNA expression of markers identifying: inducible nitric oxide synthases (iNOS), tumor necrosis factor alpha (TNF-α), monocyte chemotactic protein-1 (MCP-1), transforming growth factor beta (TGF-β), interleukin-1 beta (IL-1β), interleukin-6 (IL-6), interleukin-4 (IL-4), chitinase-like3 Chil3 (YM1), Peroxisome proliferator-activated receptor gamma (PPARg). We used the comparative Ct method for relative estimation of mRNA expression which were normalized to 18s (a housekeeping gene). To overcome the possible errors due to augmentation of contaminated DNA: (a) we used primers adjusted to bridge an intron for specific cDNA augmentation; (b) we used proper negative control reactions (template free controls); (c) we reassessed the consistency of product augmentation through examination of the melting curve of augmented products (dissociation graphs); and (d) the melting temperature (Tm) was 57°C–60°C, and the probe Tm was at least 10°C more than the primer Tm. The primer sequences are listed in **S1 Table**.

### Echocardiography

We used a Vevo 3100 system supplied with a 15-7-MHz linear broadband transducer and a 12-5-MHz phased array transducer to acquire Echocardiograms. We assessed cardiac function at baseline (before cardiac surgery) then at 48 hours after MI, and immediately before sacrifice at 30 days after MI. We utilized a heating pad to keep the body temperature at 37°C during the experiment; the temperature was measured using a rectal temperature probe. To determine the left ventricular function and volume in M-mode, two-dimensional and Doppler echocardiography modes we utilized modified parasternal long-axis and short-axis. We also used M-mode tracings at the mid-papillary level to estimate the systolic and diastolic parameters, and Teichholz formula at end-systole and end-diastole to measure the LV volumes. All mice were anaesthetized using 1%–3% isoflurane during Echocardiography to maintain a heart rate of 450–500 BPM for all echocardiographic acquisitions. Echocardiography imaging and analysis was performed by a blinded investigator.

### Cell culture and hypoxic exposure

The murine macrophage cell line J774 (ATCC, Manassas, VA) was used for the *in vitro* studies on the anti-inflammatory effects of AZM. Cells were plated in 6 well plates at concentration 0.3×10^6^ cells/well in media consisting of DMEM, 10% FBS, 1% penicillin/streptomycin, 1% sodium pyruvate, L-Glutamine, and Glucose. Following cell adhesion (4–6 hours) cells were treated with AZM (Sigma-Aldrich, St. Louis, MO) at concentration 30 μM or DMSO (SIGMA-ALDRICH, St. Louis, MO) as control and incubated over night at 37 C with 5% CO2. To induce hypoxia-reperfusion injury, cultured cells were established in a gas-tight modular chamber (STEMCELL Technologies Inc., Seattle, WA) containing 1% O2, 5% CO2, 94% N2, or in normal oxygen tension with 20% O2, 5% CO_2_ as control for 24 hours at 37°C followed by reperfusion with 21% O2, 5% CO2 for 24 or 48 hours. Supernatants were collected at the end of these time points to assess the cytokines level and their ratio (TNF-α and IL-10).

### ELISA assays

Protein concentrations of TNF-α and IL-10 are measured using standard ELISA kits (BD Biosciences, San Deigo, CA) according to the manufacturer protocol. Data are presented as individual cytokine values and their ratio.

### Luminex assay

Plasma was collected according to the above mentioned protocols at days 3, and 7 post-MI. Inflammatory biomarkers (IL-12, IL-1β, IL-1α, IL-6, TNF-α, MCP-1, MIP-1a and MIP-1b) were assayed using the Milliplex mouse cytokine magnetic kit (MILLIPLEX MAP for Luminex xMap Technology, Millipore, USA) according to the manufacturer’s protocol.

### Liver and kidney function tests

Plasma was collected according to the above mentioned protocols at days 1, 3, and 7 post-MI. Samples were sent to ANTEC diagnostic to assay blood urea nitrogen, alanine aminotransferase (ALT), albumin, and albumin/globulin ratio.

### Statistical analysis

Values are expressed as mean ± standard error of mean (SEM). We used unpaired Student t test or analysis of variance (one-way or multiple comparisons) to estimate differences, as appropriate. We utilized two-sided Dunnett or Dunn tests for post hoc multiple comparison procedures, with control samples as the control category. Throughout the analyses, a P value less than 0.05 was considered statistically significant. All statistical analyses were performed using the Prism 7 software package (GraphPad, La Jolla, CA).

## Results

### AZM shifts macrophages away from the pro-inflammatory towards the reparative state post-MI

Macrophages are the predominant inflammatory cells type in cardiac tissue after cardiac injury, regulating its healing at multiple phases [21]. It is well established that AZM can modulate inflammation through shifting macrophages towards the reparative phenotype [11–14]. To date, the effect of AZM on the inflammatory response after MI at the level of macrophages has not been characterized. We analyzed the phenotype of macrophages in cardiac tissue at several time points following induction of MI using flow cytometry (**Fig 1A**). We detected a significant reduction in the pro-inflammatory macrophages (CD45^+^/Ly6G^-^/F4-80^+^/CD86^+^) at 1 day following MI with AZM treatment (**Fig 1B**). This effect was associated with a significant increase in the reparative macrophages (CD45^+^/Ly6G^-^/F4-80^+^/CD206^+^) at day 1 and 3 after MI (**Fig 1C**). Overall, the ratio between pro- and anti-inflammatory macrophages was reversed towards an anti-inflammatory state with AZM treatment, particularly in the early phase after MI (**Fig 1D**).

**Fig 2:**
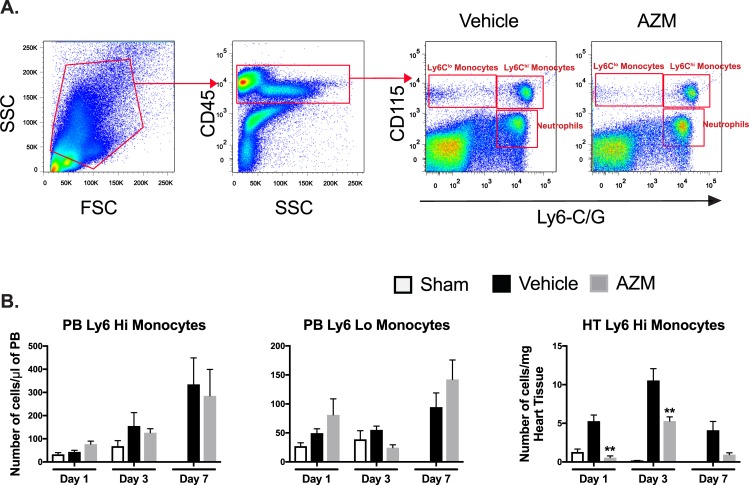
AZM therapy reduces cardiac inflammatory monocytes after MI. Representative FACS plots demonstrating the gating strategy for Ly6C^hi^ (CD45^+^/CD115^hi^/Ly6-C^hi^) and Ly6C^lo^ (CD45^+^/CD115^hi^/Ly6-C^lo^) monocytes (Panel A). Quantitative analyses of monocyte subpopulations in PB and heart tissue demonstrating no significant changes in PB monocyte subpopulations but significant reduction in cardiac Ly6C^hi^ population throughout the different time points after MI in AZM treated mice (Panel B) (n = 4 MI and 3 sham mice/group/time point, *P<0.05 and **P<0.01 compared to vehicle controls). Data presented as mean ± SEM. AZM, azithromycin; PB, peripheral blood.

Under physiological conditions, resident macrophages maintain tissue homeostasis [2]. However, after injury such as MI, monocytes infiltrate the heart and differentiate into macrophages. Monocytes contribute to tissue healing and repair through coordinated activities of different monocyte subpopulations (Ly6C^hi^ vs. Ly6C^lo^) [22, 23]. We assessed monocyte subpopulations (Ly6C^hi^ vs. Ly6C^lo^) after MI in the PB and heart (**Fig 2A**). While we did not notice significant differences between blood monocyte subsets in AZM vs. vehicle treated mice, we detected a significant reduction in the Ly6C^hi^ (pro-inflammatory) monocytes (CD45^+^/Ly6C/G^hi^/CD115^hi^) in cardiac tissue after MI with AZM treatment (**Fig 2B**). There was no significant difference in the Ly6C^lo^ (anti-inflammatory) monocytes in cardiac tissue with AZM treatment. This suggests a reduction in pro-inflammatory monocyte infiltration to the heart or a reduction in their polarization in the myocardium. Since maintained Ly6C^hi^ monocytes impair healing after MI [23], these results imply that AZM treatment reduces detrimental inflammatory response following myocardial ischemia.

### AZM downregulates pro-inflammatory cytokines while upregulating anti-inflammatory cytokines following cardiac injury

**Fig 3:**
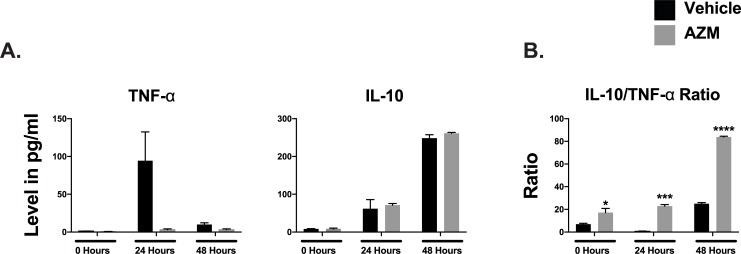
AZM therapy reduces the production of inflammatory cytokines in macrophages subjected to ischemia/reperfusion injury. Quantitative analyses of pro-inflammatory cytokine, TNF-α, and the anti-inflammatory cytokine, IL-10, production from J774 macrophages subjected to 24 hypoxia followed by 24 and 48 hours of reperfusion. The analysis demonstrates reduction of TNF- α compared to vehicle both at 24 and 48 hours. No significant changes were noted in IL-10 production. Overall, the IL-10/TNF-α ratio was significantly higher in macrophages treated with AZM (Panel B) (two independent experiments and 4 replicates/time point, *P<0.05, ***P<0.001 and **** P<0.0001 compared to vehicle controls). Data presented as mean ± SEM. AZM, azithromycin; IL-10, interleukin 10; TNF-α, tumor necrosis factor-alpha.

Macrophages have distinctive cytokine profiles based on their inflammatory status. Pro-inflammatory macrophages are potent generators of toxic effector molecules (reactive oxygen species, nitric oxide) and pro-inflammatory cytokines (IL-1β, TNF-α, IL-6) [7]. Conversely, reparative macrophages produce anti-inflammatory molecules (TGF-β and IL-10) and express scavenger, mannose, and galactose-type receptors [8, 9]. We conducted *in vitro* studies to examine the effect of hypoxia/reperfusion injury on the phenotype of J774 macrophages in the presence or absence of AZM. Following 24 hours hypoxia and reperfusion, we noted significant shift in macrophage cytokine production towards a pro-inflammatory phenotype with higher TNF-α levels compared to IL-10 levels. AZM treatment shifted the cytokine production towards an anti-inflammatory pattern with significant increase in IL-10/TNF-α ratio (**Fig 3**).

**Fig 4:**
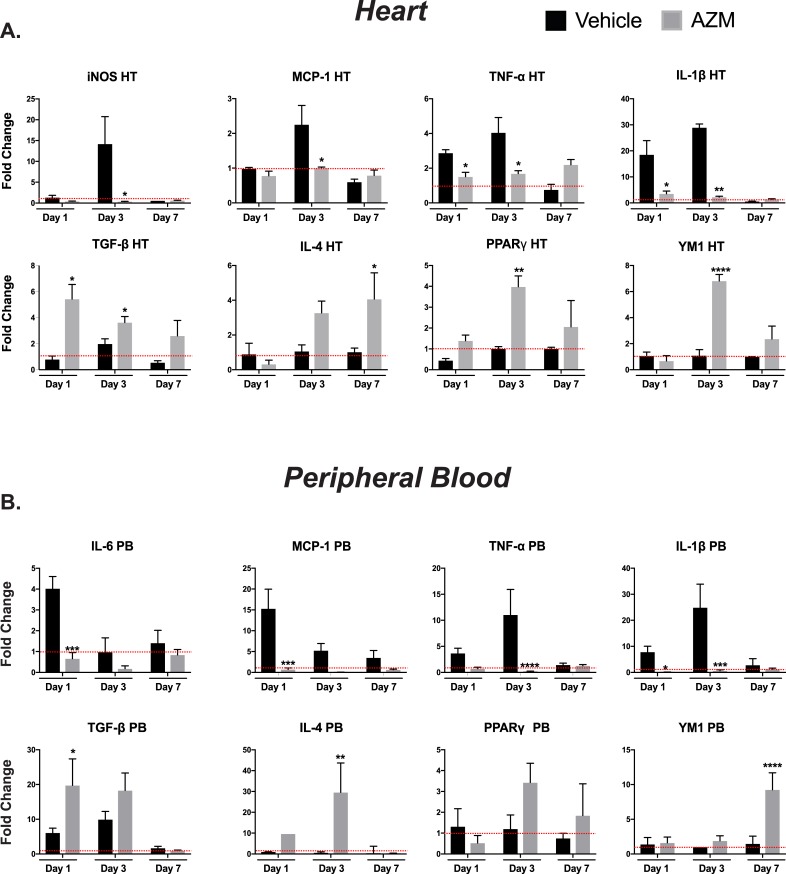
AZM treatment exerts immunomodulatory effects on cytokines expression following MI. mRNA expression of pro-inflammatory cytokines in HT (Panel A) and PB (Panel B), demonstrate significant reduction in gene expression of these cytokines in the early inflammatory phase following injury with AZM therapy compared to vehicle controls (red line demarcates the level of gene expression in sham operated mice). The mRNA expression of anti-inflammatory cytokines is augmented with AZM therapy compared to vehicle control (n = 4 mice/group/time point, *P<0.05, **P<0.01, ***P<0.001 and **** P<0.0001 compared to vehicle controls). Data presented as mean ± SEM. AZM, azithromycin; HT, heart; IL-1β, interleukin 1 beta; IL-6, interleukin 6; IL-4, interleukin 4; iNOS, inducible nitric oxide synthase; MCP-1, monocyte chemoattractant protein-1; PB, peripheral blood; PPARγ, peroxisome proliferator-activated receptor gamma; TGF-1β, tissue growth factor 1 beta; TNF-α, tumor necrosis factor-alpha; YM1 (Chil3), chitinase-like 3.

We also examined the effect of AZM on cytokine expression following MI in the *in vivo* studies. Our data confirm the change towards the anti-inflammatory state with AZM treatment. In heart tissue, AZM treatment was associated with significant reductions in the gene expression of iNOS and inflammatory cytokines (MCP-1, TNF-α, IL-6, and IL-1β) while the anti-inflammatory cytokines/macrophage modulatory factors (TGF-β and IL-4, PPAR**γ** and YM1) were increased (**Fig 4A**). A similar trend was noted in the gene expression of pro- and anti-inflammatory cytokines in PB cells at the same time points (**Fig 4B**). These gene expression results are corroborated by IHC and ELISA studies of the heart and plasma, respectively. Three days after MI, we quantified IL-1β and YM1 levels in the peri-infarct zone. IL-1β was remarkably downregulated with a concomitant elevation in YM1 in AZM treated mice compared to the control group (**S2 Fig**). In plasma, levels of inflammatory cytokines (IL-12, IL-1β, IL-1α, IL-6, TNF-α, MCP-1, MIP-1a and MIP-1b) were suppressed on days 3 and 7 post-MI as assessed by the Luminex assay (**S3 Fig**). Overall, these results imply that AZM treatment modulates the inflammatory response following myocardial ischemia by attenuating the pro-inflammatory changes and enhancing anti-inflammatory/reparative cytokine production.

### AZM treatment is associated with alternative macrophages activation in the peri-infarct region

**Fig 5:**
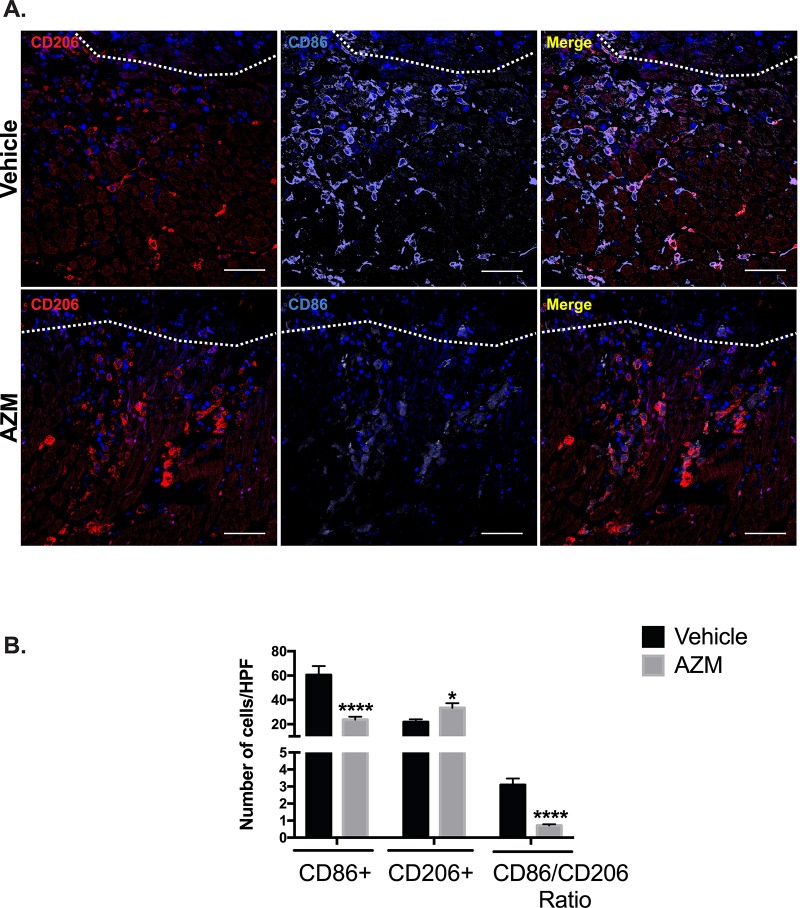
AZM treatment enhances alternative macrophage activation in the peri-infarct border of the injured heart. Immunohistochemical assessment of the content of pro-inflammatory (CD86+) and reparative macrophages (CD206+) markers 3 days post-MI. Panel A shows representative images from vehicle- and AZM-treated mice demonstrating higher density of CD86+ compared to CD206+ cells in the peri-infarct border in vehicle-treated mice. White line demarcates the infarct border. Panel B shows quantitative assessment of CD86+, CD206+ and the markers ratio 3 days post-MI. The difference in pro-inflammatory and anti-inflammatory macrophages lead to a significant shift towards an anti-inflammatory state and the reduction in their ratio in AZM-treated mice (n = 4 animals/group, *P<0.05 and ****P<0.0001 compared to vehicle controls). Scale bars represent 50 μm. Data presented as mean ± SEM. AZM, azithromycin; HPF, high power field.

The balance between pro- and anti-inflammatory macrophages in the peri-infarct region plays an important role in infarct expansion and adverse cardiac remodeling [2, 4, 21, 24]. To evaluate the impact of AZM on the activation state of macrophages in the peri-infarct region, we quantified pro-inflammatory (CD86+) and anti-inflammatory (CD206+) macrophages 3 days post-MI using immunohistochemistry. Our results confirmed the shift towards an anti-inflammatory state with AZM treatment. AZM-treated mice had significantly lower numbers of CD86+ cells and higher numbers of CD206+ cells (**Fig 5**). Importantly, the pro-inflammatory/reparative macrophage ratio were decreased robustly in the AZM treated compared to the vehicle group. Of note, macrophage inflammatory state is a spectrum from the pro- to the anti-inflammatory phenotypes. Nonetheless, we used the same strategy for assessing each population quantitatively to avoid any bias. We counted CD86 and CD206 separately and this could have led to double counting of cells in each category. These findings are in agreement with our flow cytometry data and implicate a potential therapeutic role for AZM in reducing post-MI cardiac inflammatory changes.

### AZM reduces neutrophil counts after cardiac ischemia through enhancing apoptosis

**Fig 6:**
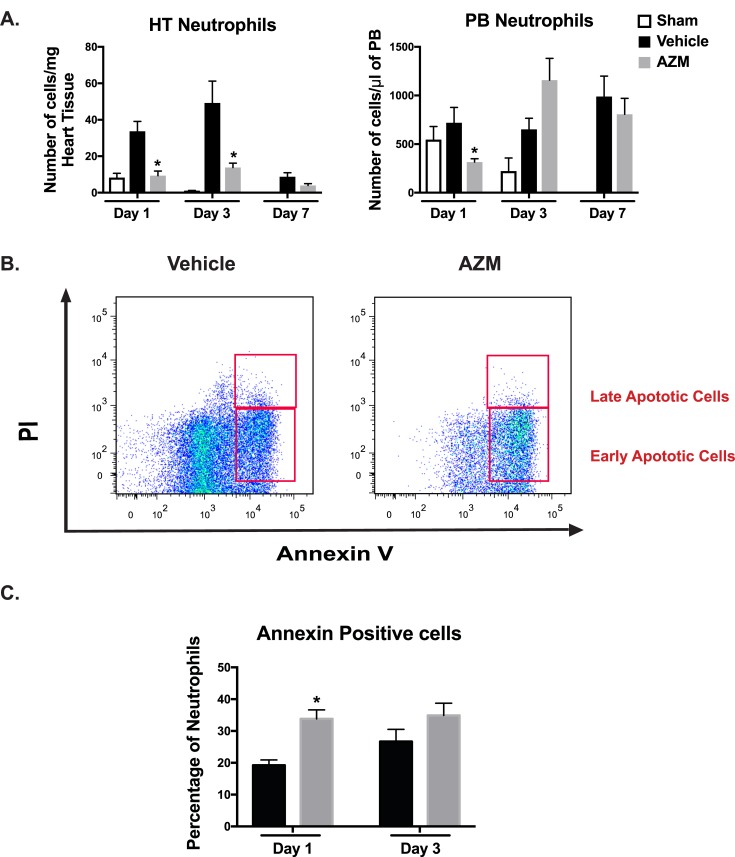
Cardiac neutrophils are reduced with AZM treatment due to apoptosis. Panel A summarizes the quantitative assessment of neutrophil (CD45 ^+^/CD115^lo^/Ly6G-C^lo^) numbers and demonstrates significant reduction in the AZM-treated group relative to controls in the heart and peripheral blood during the early inflammatory stage post-MI. Panel B illustrates the flow cytometry analyses of neutrophil populations stained against annexin V and PI showing higher percentage of early apoptotic neutrophils in AZM-treated mice. Panel C summarizes the quantitative assessment of early apoptotic neutrophils and demonstrates significantly higher percentage in the heart in the AZM-treated group during the early stage following MI (n = 4 mice/group/time point, *P<0.05 compared to vehicle controls). Data presented as mean ± SEM. AZM, azithromycin; HT, heart; PB, peripheral blood; PI, propodium Iodide.

In acute inflammation, neutrophils are key players in removing pathogens and debris, thus contributing to the return of normal tissue homeostasis [25]. Evolutionary mechanisms have developed to limit the initial inflammatory response after MI. These pathways include a robust switch of macrophages towards anti-inflammatory phenotype following ingestion of apoptotic neutrophils [26]. Indeed, prior studies have implicated the ingestion of apoptotic cells as a mechanism for the anti-inflammatory changes observed with AZM [27]. While our data as well as others have shown that AZM induces alternative polarization of macrophages towards the anti-inflammatory state, the mechanism of these changes is not entirely clear. We hypothesized that AZM promotes neutrophil apoptosis which likely initiates the anti-inflammatory changes. In support of this hypothesis, prior reports have confirmed the pro-apoptotic effects of macrolides on neutrophils [28, 29]. We therefore analyzed neutrophil numbers (CD45^+^/CD115^lo^/Ly6G/C^lo^) using the gating strategy outlined in Fig 2A in the heart and PB via flow cytometry. Treatment with AZM resulted in significant reductions of neutrophil counts in cardiac tissue and PB in the early phase after MI (**Fig 6A**). To further investigate the mechanism associated with neutrophil count reduction, we examined neutrophil apoptosis using Annexin V and PI staining in cardiac tissue using flow cytometry. AZM treatment was associated with a significant increase in the percentage of apoptotic neutrophils in the early inflammatory phase after MI compared to vehicle-treated mice (**Fig 6B and 6C**). Our findings indicate that AZM treatment reduces neutrophil numbers after cardiac injury through pro-apoptotic effects, which could account for its post-MI immunomodulatory effect.

### AZM diminishes cardiac cell death and scar size while promoting angiogenesis following cardiac ischemic injury

**Fig 7:**
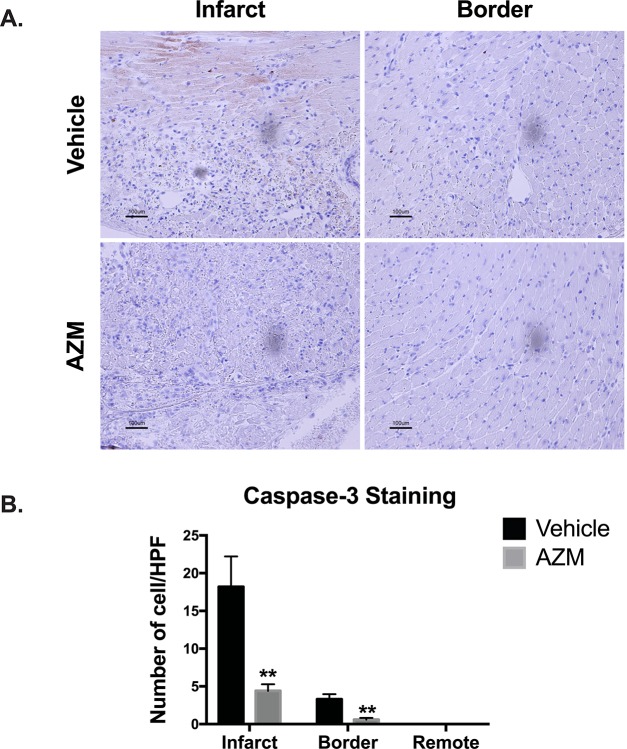
AZM reduces apoptosis post-infarction. Panel A shows representative light microscope images of cleaved caspase-3 staining for infarcted and border regions in AZM- and vehicle-treated mice 3 days after MI. Quantitative analyses (Panel B) of apoptosis reveal a remarkable reduction in caspase-3 activation in the infarct and border regions of AZM-treated group compared to the control group (n = 4 animals/group, **P<0.01 compared to vehicle controls). Scale bars represent 100 μm. Data presented as mean ± SEM. AZM, azithromycin; HPF, high power field.

**Fig 8:**
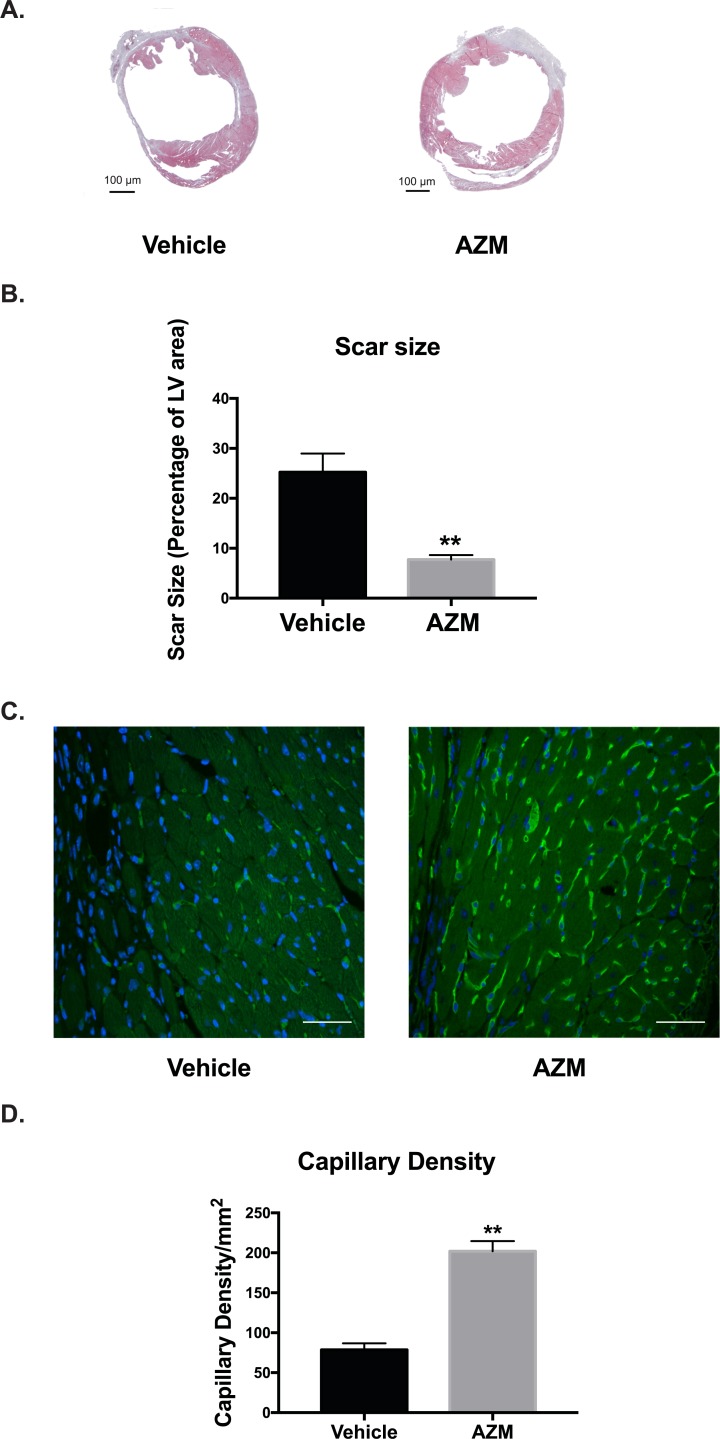
AZM treatment reduces scar size and enhances angiogenesis after myocardial injury. Representative Masson's trichrome staining at 30 days after myocardial injury in vehicle- and AZM-treated groups (Panel A). Quantitative analysis of scars as percentage of LV area shows significant reduction in AZM group relative to the control group (Panel B) (vehicle-treated, n = 15 vs. AZM-treated, n = 12, **P<0.01 compared to vehicle controls). Representative isolectin staining (Green) for capillary density in the peri-infarct region in AZM- and vehicle-treated animals demonstrates higher capillary density in AZM group compared to control (Panel C). Quantitative analysis of capillary density confirms the higher angiogenesis rate and capillary density in AZM-treated group (Panel D) (n = 4 animals/group, **P<0.01 compared to vehicle controls). Scale bars represent 50 μm. Data presented as mean ± SEM. AZM, azithromycin; MI, acute myocardial infarction; LV, left ventricular.

Alternatively activated macrophages are master regulators of the healing process after MI by regulating cardiomyocyte apoptosis, collagen deposition, and angiogenesis in the peri-infarct regions [30]. To determine the effect of AZM effects on programmed cell death in the heart post-MI, we investigated cleaved caspase-3 positive staining of cells in the infarct zone, peri-infarct border, and remote (normal) zones 3 days post-MI. We found that apoptosis was significantly reduced within the infarct and border regions in the AZM-treated group compared to vehicle-treated group (**Fig 7**). In order to confirm our caspase 3 staining results, we performed TUNEL assay in the peri-infarct region. The results were consistent with the caspase 3 assay (**S4 Fig**), suggesting enhanced survival of cardiac cells, likely driven by AZM. These data corroborate the immunohistochemistry and real-time PCR results, demonstrating significant reduction in pro-apoptotic cytokines such as TNF-α. We then assessed the effect of AZM therapy on the infarct size using morphometric assessments on Masson’s trichrome-stained cross-sections from the hearts (30 days post-MI). We observed significantly smaller scar size in mice treated with AZM compared to those treated with vehicle (**Fig 8A and 8B**). These data indicate that AZM protects the infarcted heart from the adverse cardiac remodeling that occurs chronically after ischemia.

Alternatively activated macrophages produce multiple cytokines (including pro-angiogenic factors) that can enhance regeneration [8, 9]. Angiogenesis is regarded as a major stimulator of tissue recovery in the heart after ischemic injury. To examine the vessels density in the peri-infarcted areas between groups, we assessed quantified isolectin (a marker of endothelial cells in blood vessels) 30 days following MI. Mice treated with AZM had significantly higher capillary density in the peri-infarct region compared to vehicle-treated mice (**Fig 8C and 8D**). Taken together with the results detailed in Fig 7, AZM treatment reduces scar size after MI, which could be explained by the reduction in cellular apoptosis and enhanced angiogenesis in the ischemic heart.

### AZM preserves cardiac function, reduces adverse remodeling and improves survival post-MI

**Fig 9:**
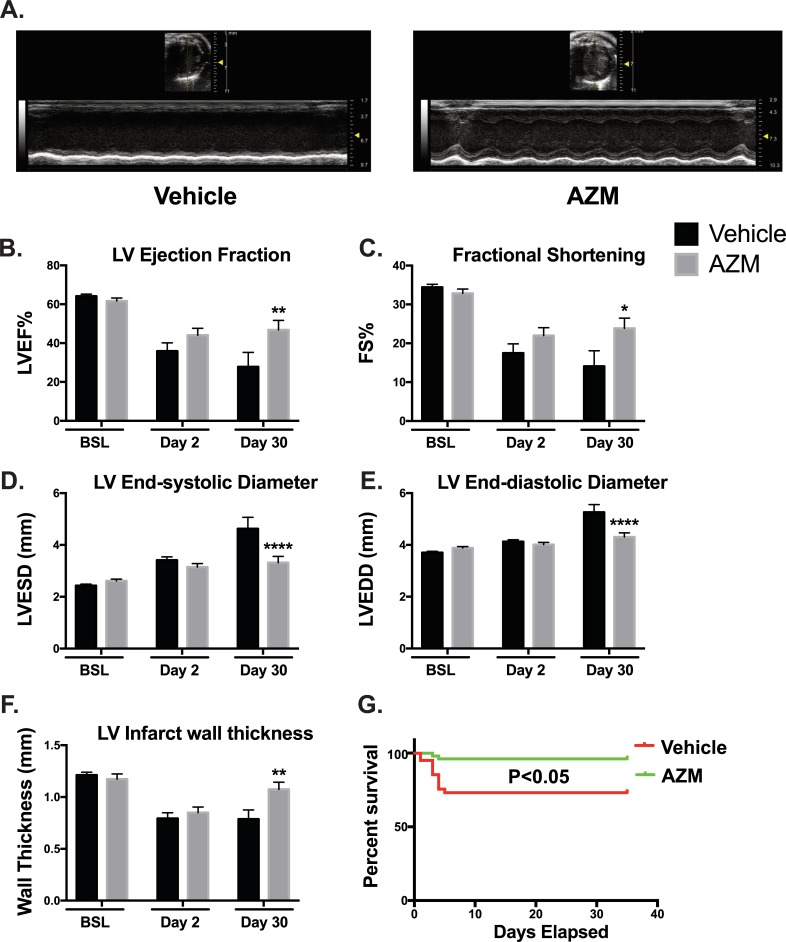
AZM treatment improves chronic cardiac remodeling and survival post-MI. 30 day following MI, transthoracic echocardiography using M-Mode (Panel A) and 2D echocardiography was performed on AZM- and vehicle-treated animals to evaluate left ventricular function and remodeling parameters. Quantitative analyses demonstrate significant recovery in LV function as assessed by ejection fraction (LVEF) (Panel B) and fractional shortening (FS) (Panel C). Data also shows significant improvements in LV adverse remodeling parameters such as end-systolic diameter (LVESD) (Panel D) and end-diastolic diameter (LVEDD) (Panel E). Additionally, we observed significantly thicker infarct walls suggesting of enhanced recovery and regeneration (Panel F). Survival curves of AZM and vehicle-treated mice 30 days post-MI demonstrate a significant improvement in survival with AZM treatment (Panel G). (Echo data: vehicle-treated, n = 15 vs. AZM-treated, n = 12; survival data: vehicle-treated, n = 48 and AZM-treated, n = 53; *P<0.05, **P<0.01 and ****P<0.001 compared to vehicle control). Data presented as mean ± SEM. AZM, azithromycin; MI, acute myocardial infarction.

Loss of balance between inflammatory and reparative phases post-MI can result in adverse cardiac remodeling and eventually systolic HF [4]. Our data suggest an immunomodulatory role for AZM on the heart post-MI. To examine the translation of these cellular effects on cardiac function, echocardiography was carried out on AZM and vehicle treated mice at baseline, and 2 and 30 days after MI. We observed preservation of LV ejection fraction as well as fractional shortening in AZM treated compared to vehicle mice (**Fig 9A–9C**). We also observed similar effects on parameters of LV remodeling such as LV end-systolic and end-diastolic diameters (**Fig 9D and 9E**). In agreement with the noted reduction in scar size, we observed significantly less deterioration in infarct wall thickness in AZM treated group compared to controls (**Fig 9F**). Therefore, the beneficial effect of AZM extends beyond the enhancements in the inflammatory balance after MI to a favorable recovery of LV functional and remodeling parameters as summarized in **S2 Table**. Additionally, survival rates reflect these AZM-mediated functional improvements with a marked reduction in mortality in AZM-treated mice (**Fig 9G**).

## Discussion

Cardiac inflammation is a crucial component of the recovery phase after MI. However, a tug-of-war between pro- and anti-inflammatory forces in the ischemic myocardium impacts cardiac recovery. Reversing the pro-inflammatory forces after MI can enhance cardiac recovery and survival. In this study, we provide first evidence that the macrolide, AZM, exerts immunomodulatory effects on macrophages after MI shifting them towards anti-inflammatory phenotype. This is demonstrated by significant reduction in pro-/anti-inflammatory macrophage ratio and pro-inflammatory genes expression in parallel with significant elevation in anti-inflammatory gene expression. These immunomodulatory effects resulted in reduced apoptosis and scar size, enhanced cardiac functional recovery, and improved survival. Taken together, AZM represents a novel and clinically available target for reducing cardiac inflammation and damage following MI.

The immunomodulatory effects of AZM after MI appear to be multifactorial including direct effects on macrophages as well as pro-apoptotic effects on neutrophils. It is well accepted that neutrophils play a critical role in tissue healing following MI through debris clearance from the injury site [31]. However, prolonged neutrophil accumulation in the heart post-MI is considered deleterious [32, 33]. On the other hand, aggressive neutrophil depletion following MI can disturb the healing process, leading to impaired functional recovery [34]. Clinical data suggest that a delay in neutrophil apoptosis after MI contributes to the exaggerated inflammatory response [35]. Here, we show that AZM enhances neutrophil apoptosis in cardiac tissue after MI, which could explain the reduction in neutrophil counts in our studies. This is in agreement with other reports in the literature that suggest a pro-apoptotic effect of macrolides on neutrophils [28, 36]. The pro-apoptotic effect of AZM is most noticeable on the first day after MI. Importantly, studies have shown that AZM is more concentrated in polymorphonuclear leukocytes, which will induce apoptosis in these cells through arresting cell cycle in G2/M [37]. Engulfing apoptotic neutrophils by macrophages stimulates the anti-inflammatory response through blocking pro-inflammatory cytokines and enhancing anti-inflammatory cytokines (IL-10 and TGF-β) and pro-resolving lipid mediators [38].

In addition to its effects on neutrophil apoptosis, AZM can have direct effects on macrophages. Our *in vitro* results indicate that AZM therapy attenuates the inflammatory changes seen in macrophages subjected to hypoxia/reperfusion injury. In addition, induction of programmed cell death in neutrophils results in decrease in the production of inflammatory cytokines and chemokines [39], as demonstrated in our study. Indeed, our experiments show that AZM treatment decreased the expression of MCP-1 which promotes Ly6C^hi^ cells recruitment to the infarcted tissue. Ly6C^hi^ monocytes differentiate into pro-inflammatory macrophages in the peri-infarct border which eventually contribute to adverse cardiac remodeling [40]. Furthermore, through the reduction in neutrophil count and modulation of macrophage phenotype, the production and deleterious effects of pro-inflammatory cytokines such as TNF-α, IL-1β, and IL-6 were diminished [41]. In parallel to the decreased pro-inflammatory cytokines, we observed significant increase in anti-inflammatory cytokines such as TGF-β and IL-4 which have been linked to favorable cardiac remodeling and functional recovery after MI [42].

The immunomodulatory effects of AZM have been demonstrated in various inflammatory and tissue injury scenarios [14, 43]. Among them, acute stroke and spinal cord injury have close resemblance to the inflammatory reactions (biphasic macrophage response and chemokine profile) happening post-cardiac injury [15, 44]. AZM treatment after spinal cord injury led to significant decrease in pro-inflammatory macrophages and increase in anti-inflammatory macrophages [13, 43]. Interestingly, and in accordance with our data, the shift in macrophage phenotype was associated with marked functional improvements in mice. The exact molecular mechanisms through which AZM exerts its immunomodulatory effects are not fully understood. However, transcription factors such as NF-kB and AP-1 are likely targets [45]. Our unpublished data suggest that AZM reduces NF-kB activation through decreasing the translocation of phospho-p65 to the nucleus. Furthermore, there is a simultaneous increase in IKKβ and IκBα with AZM therapy. This does not exclude immunomodulatory actions mediated through other intracellular signaling pathways [45].

Macrophages are essential players in the myocardial environment post-MI and their role extends beyond the inflammatory phase to tissue regeneration and healing [46, 47]. The heart possesses a remarkable regenerative capacity in the early stages of postnatal life, an ability lost by day 7 after birth [48, 49]. It has been postulated that this regenerative capacity is related to the naivety of the immune system in which macrophage modulation plays a central role [30]. Indeed, anti-inflammatory macrophages have been shown to interact with stem cells following cardiac injury leading to enhance cardiac recovery [4]. Overall, AZM therapy is associated with higher numbers of anti-inflammatory macrophages (CD206+ cells) after MI with the majority of changes occurring in the peri-infarct region as shown in our immunohistochemistry data. This phenotypic shift is associated with smaller scar, better cardiac function, and enhanced angiogenesis after MI. Additionally, we observed a significant reduction in apoptotic heart cells in both infarct and peri-infarct areas at day 3 post-MI, which could be attributed to the survival factors released from reparative macrophages [42, 50]. In summary, the early shift in macrophages towards the reparative phenotype by AZM treatment can limit acute ischemic damage and promote chronic cardiac recovery.

The balance between macrophage phenotypes is reversible [21] and can be therapeutically harnessed to enhance cardiac healing after ischemic injury. Animal studies show that early interventions aimed at altering macrophage activation state or shifting the balance towards the anti-inflammatory phenotype can substantially reduce the risk of HF development [4, 51, 52]. Kain et al initiated a phenotypic switch in macrophages after myocardial injury by using either free or encapsulated 15-epi-Lipoxin A4. They demonstrated upregulation of the reparative macrophages as well as the anti-inflammatory/macrophage modulatory genes (*Mrc-1*, *Ym-1*, *Arg-1*). Moreover, cardiac function was improved in 15-epi-Lipoxin A4 treated mice compared to controls [52]. These findings were confirmed in multiple studies using different therapeutic approaches, including formyl peptide receptor 2 [51], cardiosphere-derived cells [4], and endogenous Annexin-A1 [53]. However, studies on alternative macrophage activation in MI utilized experimental therapeutics with limited safety data in humans. Conversely, our strategy of using AZM is more clinically relevant and can be safely translated to human studies.

Healing and remodeling after MI are fundamentally regulated by the inflammatory pathways. Modulating the inflammatory response post-MI is an elusive target as studies have shown that complete inhibition of inflammation is rather harmful [54]. Indeed, therapeutic interventions targeting the systemic inflammatory pathways have yielded limited success in pre-clinical and clinical studies [54]. Delayed healing and development of ventricular aneurysm were reported with glucocorticosteroids, and their uses should be avoided post-MI [55]. On the other hand, NSAIDs use in coronary artery disease patients has been correlated with higher mortality and recurrent myocardial infarction [56]. Selective targeting of inflammatory mediators such as IL-1β using selective monoclonal antibodies demonstrated success in clinical studies, albeit with high cost and modest benefit [57]. Therefore, strategies aimed at modulating the inflammatory response rather than its suppression provide promising therapeutic modalities. In this study, we show that AZM as a small immunoregulatory molecule provides more specific modulation to the post-MI inflammation through increasing the abundance of anti-inflammatory macrophages without completely blunting the pro-inflammatory mediators.

In this study, we initiated AZM 3 days prior to MI to ensure appropriate steady-state levels at the time of injury [14]. Based on published literature [45], we anticipate rapid accumulation of AZM in macrophages and fast immunomodulatory actions. Hence, initiating AZM following MI could be beneficial as well and this is currently being studied in our lab. Another limitation in our study is the use of relatively higher dose of AZM (160mg/kg/day) than the clinically prescribed dose (10-45mg/kg) [43]. As a proof of concept, we aimed to achieve a dose consistent with our *in vitro* studies. However, multiple human studies have demonstrated AZM safety at higher doses and its wide therapeutic window. Given the allometric scaling on AZM, lower doses than those used in our study may be effective in humans [10, 47]. Finally, we sought to examine the extremes of macrophage polarization by quantifying the pro- and anti-inflammatory macrophages based on gene expression and surface markers. While this is a helpful approach for examining the effects of AZM, we acknowledge that it oversimplifies the macrophage landscape and categorizes them into only two main groups (pro- and anti-inflammatory) which may lead to overestimating either of the categories [58]. Future studies are warranted to provide a comprehensive examination of the effect of AZM on the various macrophage subtypes after MI.

### Conclusions

Cardiac inflammatory changes following MI are mediated, at least in part, by pro-inflammatory macrophages, which have detrimental effects on cardiac function and survival. This is the first study to demonstrate the efficacy of AZM as an immunomodulatory pharmacological agent for macrophages after MI. Our results indicate that the anti-inflammatory effects of AZM are related to its direct effects on macrophages and its pro-apoptotic effects on neutrophils. Systematic assessment of cardiac and systemic changes demonstrates that the beneficial effects of AZM result in significant reduction in scar size, adverse cardiac remodeling, cardiac function, and survival post cardiac ischemia. AZM has a wide therapeutic window, can be administered orally, has low side effect profile and has been approved for human use. These criteria make it an ideal agent for novel and effective therapeutic interventions to improve survival and reduce HF following MI in humans.

## Supporting information

S1 Table Forward and reverse primer sequence used in the experiments. (DOCX)

S2 Table Echocardiographic morphometric parameters at 30 days post-myocardial infarction. (DOCX)

S1 Fig AZM does not alter kidney or liver function with prolonged use after myocardial infarction. (EPS)

S2 Fig AZM treatment reduces IL-1β and increases YM1 expression in the peri-infarct border of the injured heart. (EPS)

S3 Fig AZM therapy reduces the secreted inflammatory cytokines. (EPS)

S4 Fig AZM reduces apoptosis in the infarcted heart. (EPS)

## Abbreviations

AP-1: Activator protein 1
Arg-1: Arginase 1
AZM: Azithromycin
BM: Bone marrow
CTAD: Citrate-theophylline-adenosinedipyridamole
CdCl_2_: Cadmium dichloride
CO2: Carbone Dioxide
DMSO: Dimethyl sulfoxide
ECHO: Echocardiography
ECM: Extra cellular matrix
EDTA: Ethylene diaminetetraaceticacid
ELISA: Enzyme-linked immunosorbent assay
FACS: Fluorescence-activated cell sorting
FBS: Fetal Bovine Serum
FS: Fractional shortening
G-CSF: Granulocyte-colony stimulating factor
HF: Heart failure
HPF: High power field
IKKβ: IkB kinase beta
IL-1β: Interleukin 1 beta
IL-4: Interleukin 4
IL-6: Interleukin 6
IL-10: Interleukin 10
IL-13: Interleukin 13
iNOS: Inducible nitric oxide synthase
LAD: Left anterior descending coronary artery
LIX: Lipopolysaccharide-induced CXC chemokine
LV: Left ventricular
LVEDD: Left ventricular end-diastolic diameter
LVEF: Left ventricular ejection fraction
LVESD: Left ventricular end-systolic diameter
MCP-1: Monocyte chemotactic protein-1
MMP: Metalloproteinase
MI: Acute myocardial infarction
MIP: Macrophage Inflammatory Proteins
Mrc-1: Macrophage mannose receptor 1
NF-kB: Nuclear factor-κappaB
N2: Nitrogen
O2: Oxygen
PB: Peripheral blood
PBS: Phosphate-buffered saline
PCR: Polymerase chain reaction
PI: Propidium iodide
PPARg: Peroxisome proliferator-activated receptor gamma
KCL: Potassium Chloride
PS: Phosphatidylserine liposomes
RT-PCR: Quantitative reverse transcription-polymerase chain reaction
SEM: Standard error of mean
TGF-β: Transforming growth factor beta
Tm: Melting temperature
TNF-α: Tumor necrosis factor alpha
YM1 (Chil3): chitinase-like 3
