# Supplementary material for: Azithromycin therapy reduces cardiac inflammation and mitigates adverse cardiac remodeling after myocardial infarction: Potential therapeutic targets in ischemic heart disease
Source: PLoS One. 2018 Jul 12;13(7):e0200474. doi: 10.1371/journal.pone.0200474 (PMC6042749; doi:10.1371/journal.pone.0200474)
Supplement: S1 Table — (DOCX) [file pone.0200474.s001.docx]

**S1 Table.**

| **Primer** | **Forward** | **Reverse** |
| --- | --- | --- |
| **18S** | CCAGTGGTCTTGGTGTGCTG | GGAGAACTCACGGAGGACGA |
| **i-NOS** | CAGCTGGGCTGTACAAACCTT | CATTGGAAGTGAAGCGTTTCG |
| **IL-6** | CTTCCATCCAGTTGCCTTCTTG | AATTAAGCCTCCGACTTGTGAAG |
| **MCP-1** | CCCAATGAGTAGGCTGGAGA | TCTGGACCCATTCCTTCTTG |
| **TNF-α** | TGGAACTGGCAGAAGAGG | AGACAGAAGAGCGTGGTG |
| **IL-1β** | CAACCAACAAGTGATATTCTCCATG | GATCCACACTCTCCAGCTGCA |
| **TGF-β** | CGGAGAGCCCTGGATACCACCTA | GCCGCACACAGCAGTTCTTCTCT |
| **IL-4** | ACAGGAGAAGGGACGCCAT | GAAGCCCTACAGACGAGCTCA |
| **PPARγ** | GCCAGTTTCGATCCGTAGAA | AATCCTTGGCCCTCTGAGAT |
| **Chil3 (YM1)** | AGGCTTTGCGGTCCTGAT | CCAGCTGGTGAAGTAGCAGA |
