# Supplementary material for: Azithromycin therapy reduces cardiac inflammation and mitigates adverse cardiac remodeling after myocardial infarction: Potential therapeutic targets in ischemic heart disease
Source: PLoS One. 2018 Jul 12;13(7):e0200474. doi: 10.1371/journal.pone.0200474 (PMC6042749; doi:10.1371/journal.pone.0200474)
Supplement: S2 Table — (DOCX) [file pone.0200474.s002.docx]

**S2 Table. Echocardiographic morphometric parameters at 30 days post-MI.**

| **Primer** | **Vehicle** | **AZM** | **P value** |
| --- | --- | --- | --- |
| **Left ventricular ejection fraction** | 27.9±7.4 | 46.8±4.9 | 0.04 |
| **Fractional shortening** | 14.1±4 | 23.9±2.6 | 0.049 |
| **Left ventricular end-systolic diameter** | 4.6±0.4 | 3.3±0.2 | 0.02 |
| **Left ventricular end-diastolic diameter** | 5.3±0.3 | 4.3±0.2 | 0.01 |
| **Left ventricular end-systolic volume** | 108.8±21.7 | 48.4±10.5 | 0.02 |
| **Left ventricular end-diastolic volume** | 136.2±17.8 | 84.7±8.7 | 0.01 |
| **Infarct wall thickness** | 0.78±0.09 | 1.1±0.07 | 0.02 |
